# Supplementary material for: ChAdOx1 COVID vaccines express RBD open prefusion SARS-CoV-2 spikes on the cell surface
Source: iScience. 2023 Sep 12;26(10):107882. doi: 10.1016/j.isci.2023.107882 (PMC10520439; doi:10.1016/j.isci.2023.107882)
Supplement: Document S1. Figures S1–S4 and Table S1 [file mmc1.pdf]

iScience, Volume *26*

Supplemental information

ChAdOx1 COVID vaccines express RBD open prefusion SARS-CoV-2 spikes on the cell surface

Tao Ni, Luiza Mendonça, Yanan Zhu, Andrew Howe, Julika Radecke, Pranav M. Shah, Yuewen Sheng, Anna-Sophia Krebs, Helen M.E. Duyvesteyn, Elizabeth Allen, Teresa Lambe, Cameron Bisset, Alexandra Spencer, Susan Morris, David I. Stuart, Sarah Gilbert, and Peijun Zhang

Table S1 | cryo-ET Data collection and structure determination statistics, Related to Figure 2.

	ChAdOx1 19E6			ChAdOx1 19E	
**Data collection and processing**					
Magnification		42,000 x		42,000 x	
Voltage (kV)		300		300	
Electron exposure (e–/Å^2)		~130		~130	
Defocus range (μm)		-2.5 – -6.5		-2.5 – -6.5	
Camera		K3		K3	
Energy-filter		Yes, 20 ev slit		Yes, 20 ev slit	
Super-resolution mode		Yes		Yes	
Acquisition scheme		-60°/60°, 3°, group of 3 dose-symmetric		-60°/60°, 3°, group of 3 dose-symmetric	
Frame number		10		10	
Pixel size (Å)		2.2		2.18	
No. of tilt-series		68		143	
Final particle images (no.)	28896	28896	25678	29279	29279
Symmetry imposed	C3	C1	C1	C3	C1
Map resolution (Å)	9.0	9.7	9.6	9.6	11.9
FSC threshold	0.143	0.143	0.143	0.143	0.143
B-factor sharpening	-200	-200	-200	-200	-200
Accession codes	EMD-16403	EMD-16404	EMD-16697	EMD-16405	EMD-16406
		EMPIAR-11456		EMPIAR-11457	

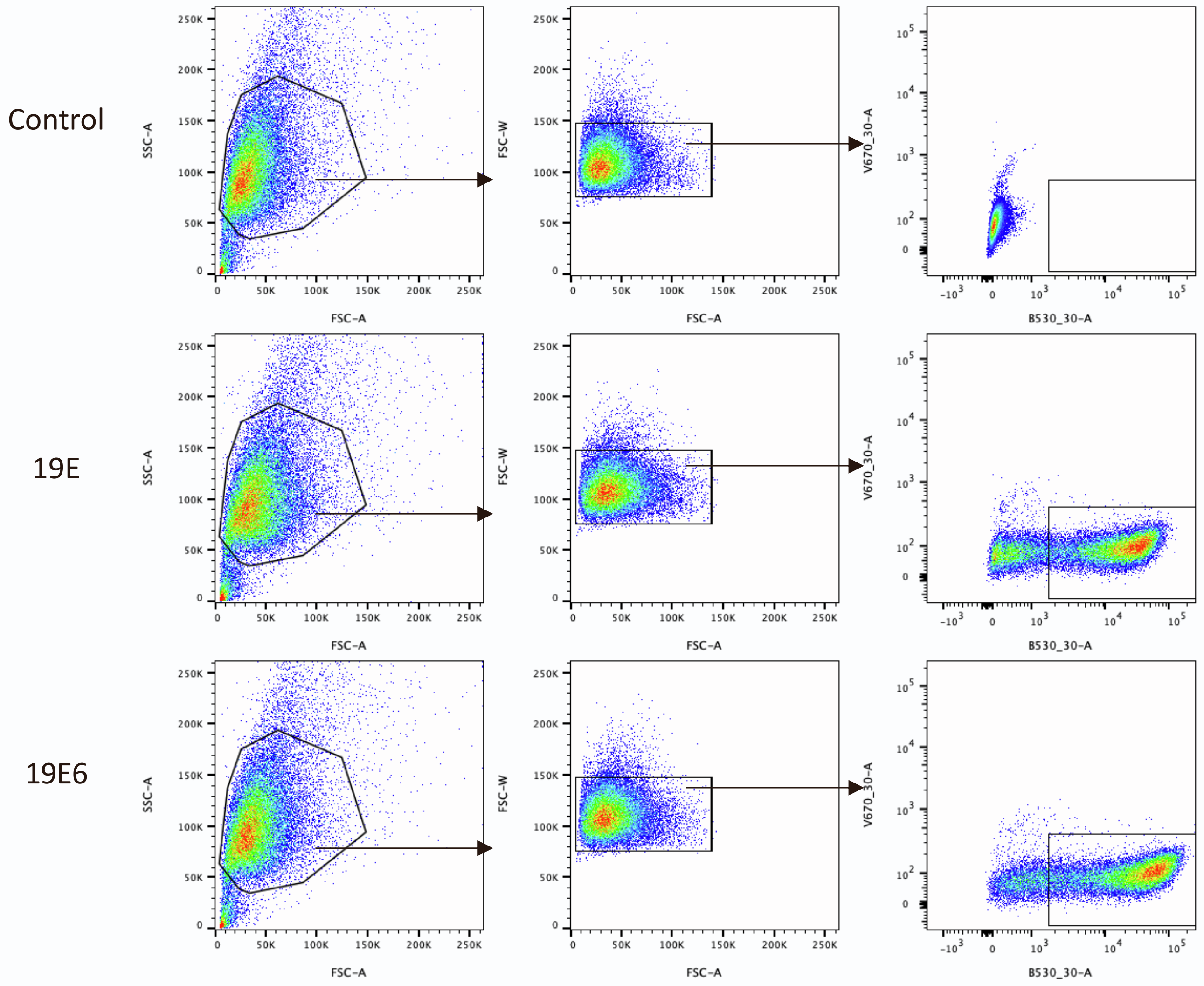

Figure S1 | Cytometry gating strategy, related to Figure 1. Cells were initially plotted according to their areas in forward and side scattering. A wide gate was used including most cells and discarding large aggregates and debri. Next, we gated on single cells on a forward scattering area vs width plot. Finally, Alexa488 positive cells were identified by plotting cells according to their intensity of Alexa 488 secondary staining (used to detect both Ab222 monoclonal antibody and ACE.Fc) vs Brilliant Violet. A third gate was used to quantify the cells with strong Alexa488 signal above the untransduced control levels.

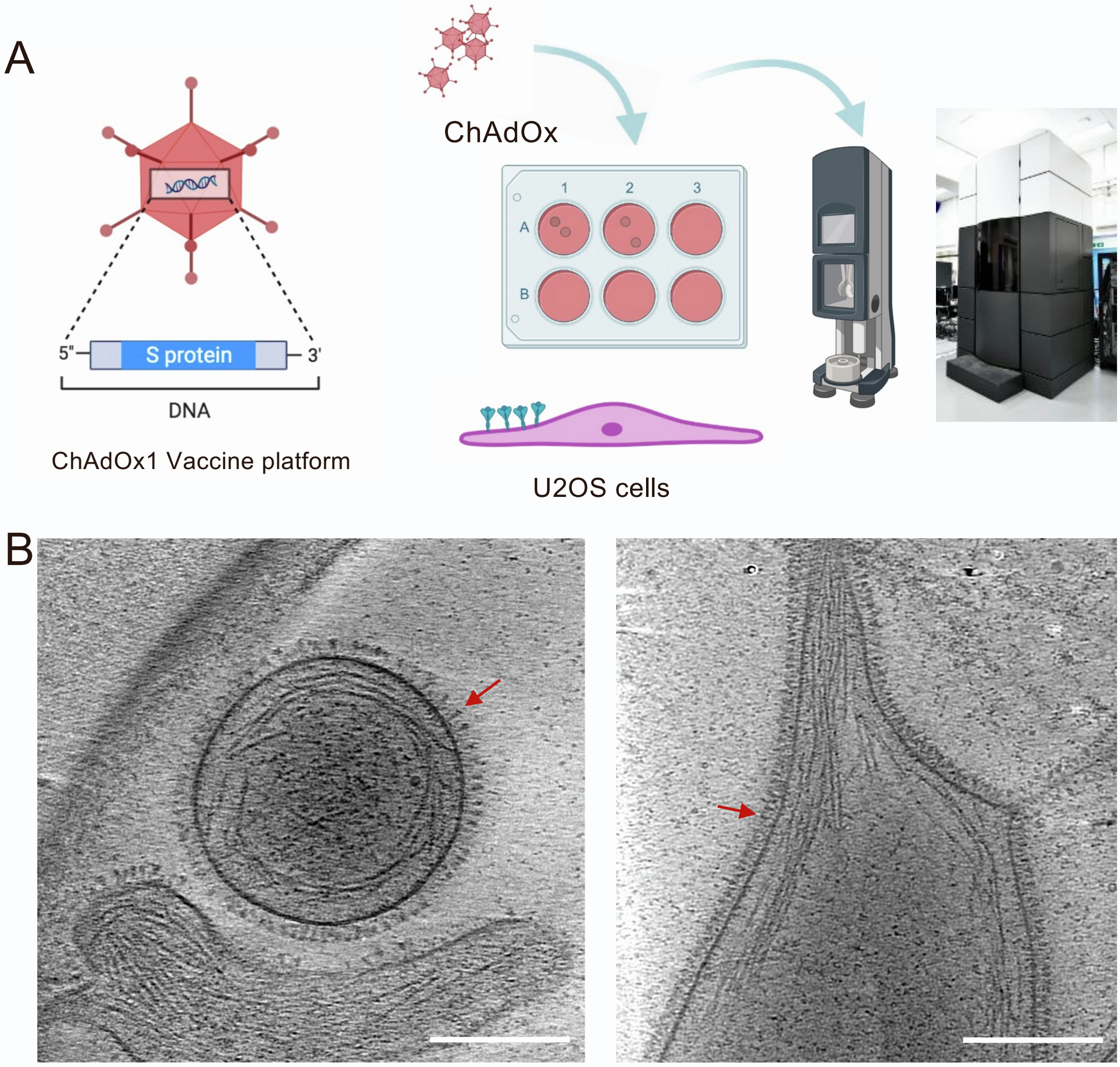

Figure S2 | CryoET analysis of ChAdOx1 vaccines, related to Figure 1. (A) Schematic of the workflow. The ChAdOx1 vectors carrying the gene encoding the Beta-variant SARS-CoV-2 spike (19E) or a HexaPro stabilized version (19E6) were used to transduce the U2OS cells grown on EM grids. Transduced cells were plunge frozen and imaged by cryoET. (B) Two representative tomogram slices of ChAdOx1 19E6 transduced cells show abundant surface expression of spikes (red arrows) on a microvesicle and cell membrane, respectively. Scale bar is 200 nm. (A and B) were created with BioRender. (A) was adapted from "COVID-19 Vaccine Candidate: AZD1222 (University of Oxford & AstraZeneca)", by BioRender.com (2020). Retrieved from https://app.biorender.com/biorender-templates

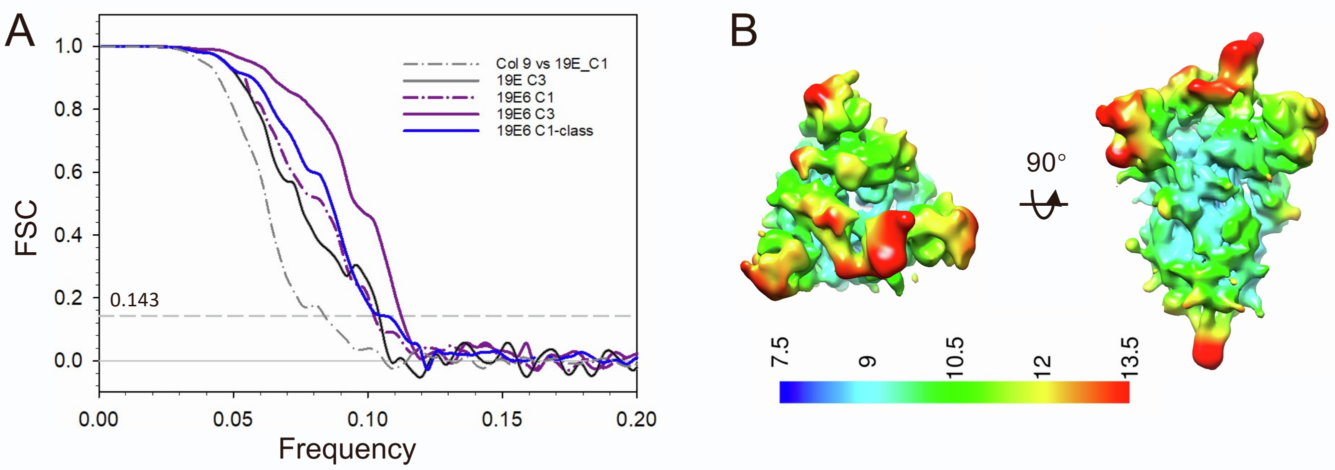

Figure S3 | Subtomogram averaging of ChAdOx1 spikes, related to Figure 2. (A) Gold-standard Fourier shell correlation (FSC) curves of subtomogram averaged ChAdOx1 19E and 19E6 spikes in C1 and C3 symmetry and 19E6 spikes after classification. (B) Local resolution of ChAdOx1 19E6 spike in C1 symmetry calculated in Relion (v4.0). Scale bar represents local resolution from 7.5 to 13.5, coloured from blue to red.

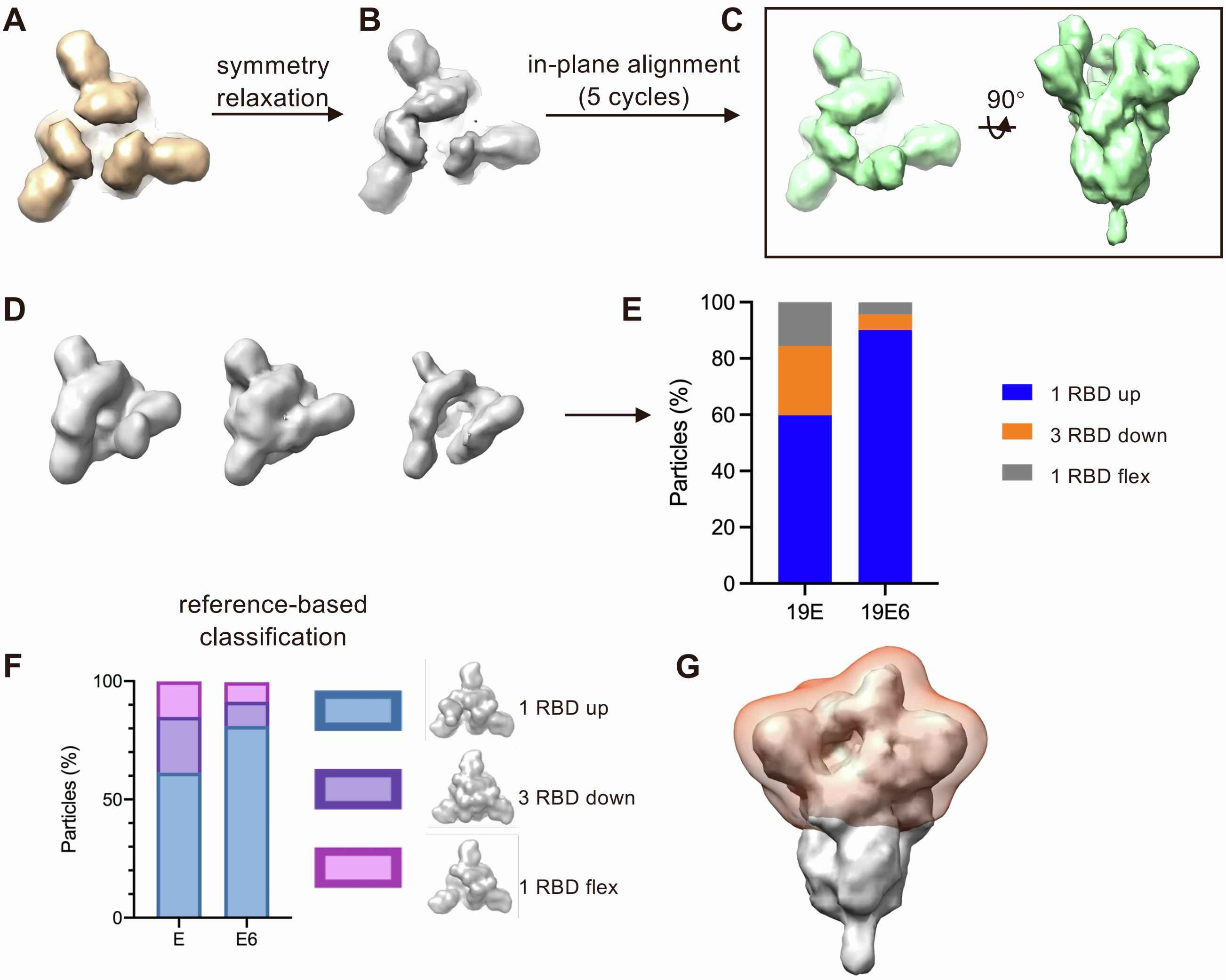

Figure S4 | Workflows of subtomogram averaging and classification of ChAdOx spikes, related to Figure 2 and 3. (A) A consensus map with 3-fold symmetry at bin2 subtomograms. (B-E) PCA-based 3D classification in emClarity. C3 symmetry is relaxed (B) and particles are aligned and refined at bin2 (C), then classified into 3 classes using PCA-based 3D classification in emClarity (D). (E) Distribution of different conformations of spikes calculated from the PCA classification method. (F) Distribution of spike conformations calculated by reference-based classification for 19E6 and WT spikes. The references were generated in Chimera using three different PDBs and imported to emClarity. Each particle is aligned to these references and classified into one of these three classes based on the highest cross-correlation coefficient score. (G) Overlay of a spike density map with the molecular mask (semi-transparent, orange) which was used for 3D classification.
